# Inclusion of non-inferiority analysis in superiority-based clinical trials with single-arm, two-stage Simon's design

**DOI:** 10.1016/j.conctc.2020.100678

**Published:** 2020-11-28

**Authors:** Miguel Sampayo-Cordero, Bernat Miguel-Huguet, José Pérez-García, David Páez, Ángel L. Guerrero-Zotano, Javier Garde-Noguera, Elena Aguirre, Esther Holgado, Elena López-Miranda, Xin Huang, Andrea Malfettone, Antonio Llombart-Cussac, Javier Cortés

**Affiliations:** a Medica Scientia Innovation Research (MedSIR), Barcelona, Spain; b Hospital Universitari de Bellvitge, L'Hospitalet, Barcelona, Spain; c IOB, Institute of Oncology, QuironSalud Group, Barcelona and Madrid, Spain; d Hospital de la Santa Creu i Sant Pau, Universitat Autònoma de Barcelona, Barcelona, Spain; e Vanderbilt-Ingram Cancer Center; Vanderbilt University Medical Center, Nashville, TN, USA; f Hospital Arnau de Vilanova, Valencia, Spain; g Hospital QuironSalud Group, Zaragoza, Spain; h Ramón y Cajal University Hospital, Madrid, Spain; i Pfizer Global Research and Development, La Jolla, USA; j FISABIO - Hospital Arnau de Vilanova, Valencia, Spain; k Vall d'Hebron Institute of Oncology (VHIO), Barcelona, Spain; l Medica Scientia Innovation Research (MedSIR), Ridgewood, NJ, USA

**Keywords:** Non-inferiority, Switching to non-inferiority, Two-stage, Single-arm, Phase II, Group sequential designs

## Abstract

**Introduction:**

Non-inferiority (NI) analysis is not usually considered in the early phases of clinical development. In some negative phase II trials, a post-hoc NI analysis justified additional phase III trials that were successful. However, the risk of false positive achievements was not controlled in these early phase analyses. We propose to preplan NI analyses in superiority-based Simon's two-stage designs to control type I and II error rates.

**Methods:**

Simulations have been proposed to assess the control of type I and II errors rates with this method. A total of 12,768 two-stage Simon's design trials were constructed based on different assumptions of rejection response probability, desired response probability, type I and II errors, and NI margins. P-value and type II error were calculated with stochastic ordering using Uniformly Minimum Variance Unbiased Estimator. Type I and II errors were simulated using the Monte Carlo method. The agreement between calculated and simulated values was analyzed with Bland-Altman plots.

**Results:**

We observed the same level of agreement between calculated and simulated type I and II errors from both two-stage Simon's superiority designs and designs in which NI analysis was allowed. Different examples has been proposed to explain the utility of this method.

**Conclusion:**

Inclusion of NI analysis in superiority-based single-arm clinical trials may be useful for weighing additional factors such as safety, pharmacokinetics, pharmacodynamic, and biomarker data while assessing early efficacy. Implementation of this strategy can be achieved through simple adaptations to existing designs for one-arm phase II clinical trials.

## Highlights

• Inclusion of non-inferiority analysis in single-arm trials is useful for weighing activity with safety and other data.
• This strategy has been previously used in proof-of-concept studies to design successful randomized phase III trials.
• However, it was a post-hoc analysis to deal with negative findings in phase II trials. So, the type I error was inflated.
• We propose to plan the non-inferiority analysis in the study protocol to control false positive findings.
• It can be made through simple adaptations to the Simon's two-stage design and other existing designs for single-arm trials.

## Abbrevations

α: The planned level of type I error
1 ‒ β: The planned level of power
a: The prespecified superiority efficacy boundary
a_ni_: The prespecified no-inferiority efficacy boundary
CI: Confidence interval
FDA: Food and drug administration
M1: The assumed effect of the active control against placebo
M2: The largest loss of effect that would be clinically acceptable
n: The number of patients the end of the trial
n1: The number of patients at the interim analysis
NI: Non-inferiority
NIM: Non-inferiority margin
p: The effect of the test drug
p0: The effect of the drug under the null hypothesis (or the effect of the active control therapy)
p1: The effect of the drug under the alternative hypothesis
RR: Response rates
UMVUE: Uniformly Minimum Variance Unbiased Estimator

## 1 Introduction

A single-arm phase II trial is the proof-of-concept stage in drug development, and focuses on the evaluation of new therapeutic hypothesis and strategies in a clinical setting [1,2]. Phase II studies in oncology are often multistage trials. Two-stage designs are becoming increasingly more common, allowing for early trial termination in cases with low response rates (RRs) towards avoiding wasting time resources on ineffective treatments. These trials aim to determine whether the new regimen is superior to a pre-specified RR (often 5%) or experience with the standard of care, whereas the alternative hypothesis is that RR is somewhat higher, say 20% [3,4].

Nevertheless, the non-inferiority (NI) question might also be relevant in the phase II setting [5]. A typical scenario is one in which an experimental treatment is potentially less toxic, less costly, easier to administer or with novel biological pathways than a conventional treatment, but these do not represent a reduction of efficacy or in percentage of patient with clinical benefit [6].

In accordance with the European Medicines Agency guidelines [7], in any superiority trial where NI may be an acceptable outcome, it is prudent to specify a NI margin in the protocol to avoid serious difficulties that can arise from later designation. Specification of a margin after viewing the results can produce an increase in the alpha error rate [8]. In 2016, the Food and Drug Administration published guidelines to establish effectiveness in NI trials. The statistical issues associated with NI studies, and procedures used to determine the NIM, have been extensively described [6].

Among all available multistage designs, the most popular is a two-stage design with a futility stopping point based on Simon's minimax or optimal criterion [9]. The simplicity of Simon's design may account for its popularity. However, the inference procedures used in two-stage designs are often not corrected to account for these designs' adaptive nature [10]. For point estimation, previous authors have developed a method to calculate the Uniformly Minimum Variance Unbiased Estimator (UMVUE) for Simon's designs and to achieve optimal results. What is more, p-values and type II errors can be calculated with stochastic ordering of the UMVUE [9,11]. These methods can be used when the realized sample size at the stopping stage is different from that specified in the initial design, and this property makes them very useful for designing and analyzing two-stage phase II trials [9].

The aim of the present study is to assess the validity of the UMVUE-based calculation method planning two-stage Simon's design phase II trials with a superiority analysis, where NI analysis is also allowed.

## 2 Methods

### 2.1 NI analysis

In a NI analysis, the goal of the study is to show that the effect of the test drug (p) is not inferior to the effect of the active control (p_0_) by a specified amount, called NIM. The null and alternative hypotheses should be defined as follows [6]:

(1) H_0_: p_0_ – p ≥ NIM (p is inferior to the control (p_0_) by NIM or more);

(2) H_a_: p_0_ – p < NIM (p is inferior to the control (p_0_) by less than NIM).

A challenging point in NI analysis is to distinguish an effective treatment from a less effective or ineffective treatment. The presence of assay sensitivity in a NI trial should be stated from: i) historical evidence of sensitivity to drug effects based on well controlled trials and a robust statistical and clinical judgment (e.g. A treatment cannot be used as a control arm if the superiority against placebo was inconclusive in historical studies); and ii) appropriate conduct of the trial that adheres closely to the design of the trials used to determine that historical evidence of sensitivity to drug effects exists [12]. The margin chosen for a NI trial should be defined prior to study initiation, taking into these historical evidences. Although the NI margin used in a trial can be no larger than the entire assumed effect of the active control against placebo (M1), it is generally desirable to choose a lower margin (M2) that reflects the largest loss of effect that would be clinically acceptable [6]. Showing NI to M1 provides assurance that the test drug had an effect greater than zero, but, in many cases, that is not sufficient to conclude that the test drug had a clinically acceptable effect [6]. In a fixed margin approach, the NIM could be considered as the risk ratio or risk difference, reflective of the average effect of the active control over placebo in previous studies [(p_control_/p_placebo_) > 1 or (p_control_ - p_placebo_) > 0], for example:

(3) Relative risk = 2.64, 95% confidence interval (CI): (1.72 to 3.56).

(4) Risk difference = 0.15, 95% CI: (0.07 to 0.22).

We selected the 95% CI lower bound (1.72 or 0.07) and adjusted to retain at least 50% of the historical effect of active control versus placebo arm ([1.72^(1-0.5) = 1.31] or [0.07*(1-0.5) = 0.035]) [6]. Accordingly, the calculated NIM describes a ratio or a difference reflecting the largest loss of effect in control group RR (p_0_) that would be clinically acceptable. Therefore, the null and alternative hypothesis of NI analysis can be defined as follows and depending on p_0_/NIM:

(5) H_0_: p ≤ (p_0_ / NIM_as ratio_) or H_0_: p ≤ (p_0_ - NIM_as difference_);

(6) H_1_: p > (p_0_ / NIM_as ratio_) or H_1_: p > (p_0_ - NIM_as difference_);

Risk ratio is preferred because it is less affected than risk differences by variability in the event rates in the placebo group [6].

### 2.2 Include a NI analyses in a superiority based single-arm design

In a superiority analysis design with tumor response as the primary endpoint, analyze firstly a NI hypothesis does not inflate the type I error rate when NI analysis and NIM are properly pre-specified [7]. Additionally, the final number of responders needed to achieve the NI objective will always equal or lower than the prespecified superiority efficacy boundary (a) [7].Therefore, we assumed the same number of patients as in superiority analysis (n); and a_ni_ (number of responding patients in NI analysis) is chosen as the lowest integer satisfying the type I error rate in NI analysis (α_ni_) ≤ α:

(7) B(a_ni_|n, p_0_ / NIM) ≥ 1- α.

The power should be calculated:

(8) 1 - β_ni_ = B(a_ni_ −1|n, p_0_/NIM); where a_ni_ ≤ a; 1 - β ≤ 1 - β_ni_.

Accordingly, the study will achieve a positive finding when “p” is equal or higher than “p_0_/NIM” and significance level evaluated by binomial test in NI analysis is ≤ α. As the NI analysis has the same expected accrual and lower or equal number of responders needed to declare significance than superiority analysis (a_ni_ ≤ a), power always will be equal or greater in NI than superiority criteria. Thus, this design can assess superiority and NI criteria with the same sample size, type I and type II error rates used in the superiority strategy (as outlined in the Supplementary Methods) [7].

Additionally, in a single-arm two-stage Simon's design, UMVUE-based calculation of p-value is still valid when the realized sample size and number of responders to achieve a positive finding are different at the stopping stage from that specified in the design (as outlined in the Supplementary Methods) [9]. Thus, include a NI strategy in a superiority based single-arm trial may be implemented in Phase II Simon's designs.

A numerical example has been proposed in Results section.

### 2.3 Simulations

A total of 12,768 two-stage, single-arm designs were computed based on different assumptions of p_0_, p_1_, α_1_, 1 - β. The package “Clinfun” (function “ph2simon”) from R software [13] was used for computing these designs [14].

The NIMs selected to formulate the rejection proportion ranged between 1 and 1.45 in 0.05 increments [8]. P-values and type II errors in every design were calculated with stochastic ordering of UMVUE [9]. A user-defined function in the R software was used for calculating these P-values and type II errors. Alpha and beta errors were simulated with the Monte Carlo method. Number of random samples generated were based on the need to attain 95% confidence, so that simulated values of alpha and beta errors were within 0.5% and 1% of the true values, respectively [15]. Agreement between calculated and simulated values were analyzed with Bland-Altman plots (as outlined in the Supplementary Methods) [16,17]. The R code detailing this process and illustrating the simulations can be found in the Supplemental Files 1 and 2. The datasets generated in these analyses can be found in supplemental datasets 0, 1, and 2. A summary detailing the information reported in these supplementary files can be found in the SupplementaryDataSummary document.

## 3 Results

### 3.1 Agreement between simulated and calculated statistical errors

**Fig. 1:**
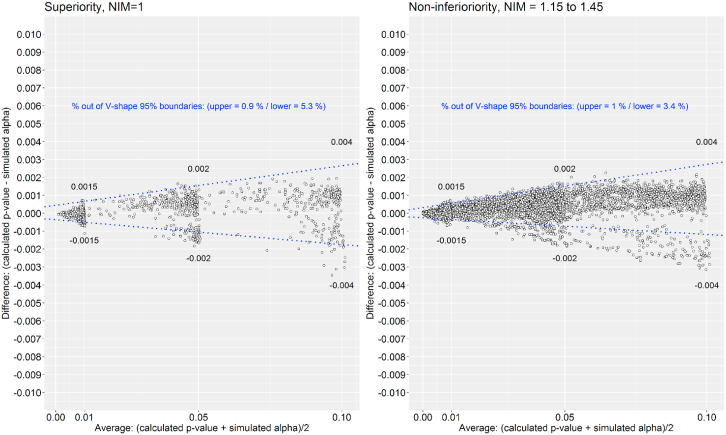
Agreement between calculated p-value and simulated alpha errors in Simon's two-stage clinical designs.

The results showed a proportional bias between calculated p-values and simulated alpha levels. Higher levels of type I error related to greater absolute differences between the calculated p-values and simulated alpha errors. This is not surprising considering that high statistical error is likely reflective of small sample size and high imprecision. Moreover, we observed that the lower boundary of the 95% CI was crossed by more than 2.5% of the differences. This finding suggests that calculated p-values tended to be slightly lower than the simulated alpha values (Fig. 1).

**Fig. 2:**
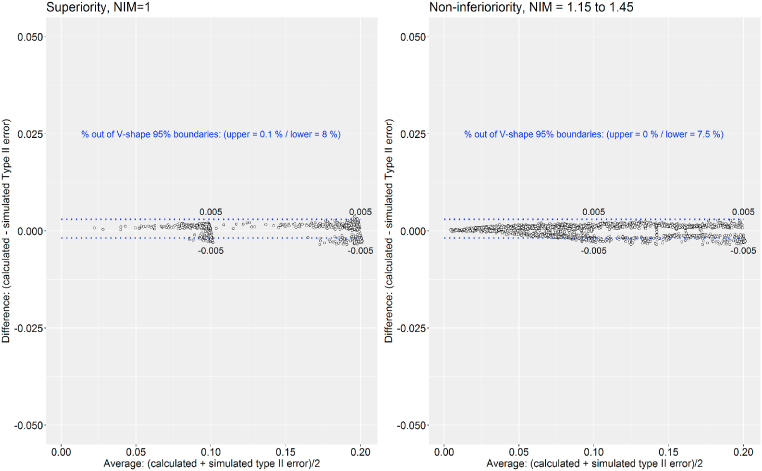
Agreement between calculated and simulated type II error in Simon's two-stage clinical designs.

However, it is important to consider that these two biases are common in superiority designs with (NIM>1) and without (NIM = 1) NI analyses. Additionally, the percentage of values crossing the 95% CI boundary is equivalent in both designs (Fig. 1). Comparison of type II errors also reflected that calculated type II errors tended to be slightly lower than the simulated type II errors. However, superiority design with and without NI strategy displayed equivalent results with about 95% limits of agreement (Fig. 2).

In the superiority scenarios (NIM = 1), we observed that the cloud of points was grouped in values of 0.01, 0.05, and 0.1 for the p-value, and 0.1 and 0.2 for the type II error. This is consistent with the pre-specified design constrains. However, we did not observe this behavior in scenarios where NIM >1, because the designs’ sample sizes were pre-specified for superiority analysis. So, type I and II errors that fulfilled design constraints for different values of NIM showed higher variability. Moreover, there were seven times more scenarios in NIM >1 analysis than in superiority analysis (NIM = 1).

The maximum differences between calculated p-values and type I errors were 0.0015, 0.002, and 0.004 for 0.01, 0.05 and 0.1 type I errors, respectively (Fig. 1). Therefore, in a study with a 0.01 significance level, the maximum simulated type I error ranged from 0.0085 to 0.0115. Studies with a 0.05 significance level had maximum simulated type I errors ranging from 0.048 to 0.052. Finally, studies with 0.1 significance levels had maximum simulated type I errors that ranged from 0.096 to 0.104 (Fig. 1).

Regarding beta errors, the maximum bias in designs with 0.1 and 0.2 type II errors was 0.005, respectively. Therefore, calculated type II errors ranged from 0.195 to 0.205 and from 0.095 to 0.105 for designs with 80% and 90% of simulated power, respectively (Fig. 2). These results suggest that differences between calculated and simulated scores are not relevant.

Collectively, our findings implied that the minimum and maximum differences for calculated and simulated values were equivalent in superiority (NIM = 1) and NI (NIM > 1) scenarios (Figs. 1 and 2). Additionally, results suggest that the agreement between calculated and simulated statistical errors decrease the greater the error tolerated in the statistical design and the calculated statistical errors tend to be lower than simulated ones. Therefore, it would not be recommended to plan single-arm designs with error constraints more relaxed than usually accepted levels (alpha and beta errors equal or lower than 0.2) [18].

Absolute differences have been plotted against average of calculated and simulated scores. The type I errors values considered were 0.1, 0.05 and 0.01, and type II errors values were 0.2 and 0.1. The NIMs selected to formulate the rejection proportion (p0/NIM) were (1, 1.15, 1.2, 1.25, 1.30, 1.35, 1.4, and 1.45). A maximum of 2.5% deviation defined the 95% limits of agreement.

Absolute differences have been plotted against average of calculated and simulated scores. The type I values considered were 0.1, 0.05 and 0.01, and type II errors were 0.2 and 0.1. The NIMs selected to formulate the rejection proportion (p0/NIM) were (1, 1.15, 1.2, 1.25, 1.30, 1.35, 1.4 and 1.45). A maximum of 2.5% deviation defined the 95% limits of agreement.

### 3.2 Numerical example

We suggest to use a single-arm trial designed to assess a new investigational therapy combined with an approved drug which represents the standard of care [19], or a dose modification of standard of care aiming to reduce the probability of related adverse events and improve quality of life [20,21]. The safety profile improvement of the standard of care will only be acceptable if it is accompanied by a NI result evaluating the treatment activity. So, it makes sense to propose a NI contrast for the evaluation of early efficacy. In addition, the reduction of toxicity could increase adherence to treatment, so the analysis of efficacy in terms of superiority should be also considered. This strategy allows us to better weigh the risk-benefit ratio of this intervention compared with the unique analysis of the safety profile. Our proposal explains how to pre-plan this study design to control type I error and avoid the post-hoc data review [6].

We designed a proof-of-concept study to evaluate if the new investigational therapy is promising, with less than 30 patients recruited. The sequence of the analysis at the end of study firstly assessed the safety profile, then the NI efficacy objectives, and finally, the superiority efficacy objective. In accordance with fixed sequence method, the NI and superiority analyses only could achieve a positive finding if previous assessment met the requirement for statistical significance [22]:

1) Firstly, we assumed that the safety objective will be achieved at the end of the study. On the contrary, if the safety objective had not been reached, the efficacy analyzes would not make sense.
2) Secondly, we planned the statistical design for early efficacy objective based on two-stage Simon's design for superiority analyses. We planned a 35% historical response rate in standard of care. This response rate could range between 30% and 40% in accordance with the baseline patients' characteristics (e.g.: prior therapies received, or prior lines of therapy) [24].

Thus, rejecting a 20% rate of responders would be a conservative approach to assess this therapy (null hypothesis). Moreover, we planned to detect an improvement greater than or equal to 45% (which represented the alternative hypothesis). We proposed an optimal two-stage Simon's design with the same error constraints accepted for the safety objective (α = 0.1 and 1 ‒ β = 0.9). For the design parameters:

(9) (p_0_, p_1_, α*, 1 ‒ β*) = (0.20, 0.45, 0.1, 0.9)

The optimal design was given by:

(10) (a_1_ / n_1_, a / n) = (4 / 14, 8 / 25)

where a1, n1, a, and n are the number of responders needed to move to the second stage, the number of accrued patients at first stage, the number of responders to achieve a positive result at final analysis, and the total number of patients recruited, respectively. Calculations were implemented in the R “Clinfun” library (function “ph2simon (0.20, 0.45, 0.1, 0.1)”) [14].

3) Thirdly, we calculated the number of responders who were needed to achieve a significant result at final analysis based on the NI hypothesis. We assumed a NIM of 1.2. The number of patients recruited in each stage and interim boundaries (a_1,_ n_1_, and n) were the same than previous superiority design. However, the number of responders to achieve a positive result in the final analysis were reduced:

(12) (a1 / n1, a / n) = (4/ 14, 7 / 25)

The calculation of p-value and 95% confidence intervals were implemented in the R “clinfun” or “OneArmPhaseTwoStudy” library [11,28].

In addition, three potential scenarios of analyses describe why our proposal is more informative than the usual alternatives, as follows:

• Scenario 1:

At the end of recruitment, a common issue is that clinical trials passed partially the pre-designed number of recruited patients. In accordance, our study achieved the interim objective to continue to the second stage (with at least 4 responders) and passed the target sample size of two patients (N = 27) at final analysis. The total number of responders observed at the end of study was 7 (x = 7). However, according to the expected sample size, in the first 25 recruited patients only 6 responses were observed. Analyzing final data with the UMVUE based method allowed to analyze all the patients recruited in the study and account the adaptive nature of the study due to the interim futility analysis [9,27]. The results based on the UMVUE method estimated a 32.2% rate of responders. The p-values for the NI and superiority analysis were 0.097 (<0.1 significant criteria achieved) and 0.194 (>0.1 non-significant), respectively. These calculations could be performed in open-access statistical libraries (packages “clinfun” or “OneArmPhaseTwoStudy”, R statistical software) [11,28]:

Package “clinfun”:

(13) NI analysis: twostage.inference(x, (a1-1), n1, n, p_0_/NIM)

(14) twostage.inference(7, 3, 14, 27, 0.2/1.2)

(15) Superiority analysis: twostage.inference(x, (a1-1), n1, n, p_0_)

(16) twostage.inference(7, 3, 14, 27, 0.2)

Package “OneArmPhaseTwoStudy”:

(17) NI analysis: get_p_KC(x, (a1-1), n1, n, p_0_/NIM)

(18) get_p_KC(7, 3, 14, 27, 0.2/1.2)

Superiority analysis: get_p_KC(x, (a1-1), n1, n, p_0_) (19)

get_p_KC(7, 3, 14, 27, 0.2) (20)

Alternatively, we could analyze results based on the pre-designed sample size boundaries. We considered the first 25 patients with only 6 responders (<7 responders, so the result was non-significant). Moreover we could conduct the analysis based on maximum-likelihood method without including the interim decision in the analysis [9]. This method only considers the total number of responders (x = 7) and the total number of patients recruited (n = 27) (function “binom.test”, package “stats”, R statistical software). We observed that the maximum likelihood method underestimated the rate of responders (25.9% < 32.3%) and did not declare significant differences neither in NI (p-value = 0.151) nor superiority analyses (p-value = 0.287). As example in R code:

NI analysis: binom.test(x = 7, n = 27, p = 0.2/1.2, alternative = 'greater') (21)

(22) Superiority analysis: binom.test(x = 7, n = 27, p = 0.2, alternative = 'greater')

However, a significant reduction in the rate of clinically relevant adverse events with a 32.2% response rate was a promising result to design a randomized trial. This result probably would have led to the decision to develop the randomized clinical trial even if the efficacy NI analysis was not formally considered. Whenever efficacy is ranked with safety, quality of life, cost or any other endpoints, efficacy is always evaluated in terms of NI among standard of care [25,[29], [30], [31]]. Nevertheless, it was not formally defined and this post-hoc analyses usually increase type I error [6].

• Scenario 2:

We would like to propose a scenario whose patients’ recruitment is discontinued prior to reach the pre-designed sample size. The study started the stage II, but it is finally discontinued with 6 responders and a total of 20 patients recruited. Reasons for the study discontinuation were not due to safety or efficacy results but to external issues (e.g. a logistic problem or a global pandemic) [32]. If we analyze these data based on the pre-designed sample size boundaries, we cannot conclude if the experimental therapy is promising or not. The number of responders was lower than efficacy boundary (≥7 responders), but we cannot reject that the boundary will be achieved with a higher number of patients recruited. However, if we based the test in the UMVUE method, the analysis could be conducted with a different number of patients than expected in the sample size [9]. Results based on the UMVUE method estimated a 32.9% rate of responders. The p-values for the NI and superiority analysis were 0.085 (<0.1 significant criteria achieved) and 0.167 (>0.1 non-significant), respectively. As example:

(23) NI analysis: twostage.inference(x, (a1-1), n1, n, p_0_/NIM)

(24) twostage.inference(6, 3, 14, 20, 0.2/1.2)

(25) Superiority analysis: twostage.inference(x, (a1-1), n1, n, p_0_)

(26) twostage.inference(6, 3, 14, 20, 0.2)

Alternatively, the maximum likelihood method underestimated the rate of responders (30% < 32.9%) and did not declare neither significant differences in NI (p-value = 0.102) nor superiority analyses (p-value = 0.199). As example:

NI analysis: binom.test(x = 6, n = 20, p = 0.2/1.2, alternative = 'greater') (19)

Superiority analysis: binom.test(x = 6, n = 20, p = 0.2, alternative = 'greater') (20)

Moreover, regarding the scenario previously reported, a significant reduction in the rate of adverse events with a 32.9% response rate represented a promising result to design a future randomized trial.

• Scenario 3:

It could be argued that designing a clinical trial with a NI analysis is unnecessary because a more conservative null hypothesis (16.7%) could be assumed, and the same result should be obtained. However, considering this alternative, the superiority objective could not be assessed, such as during a dose-finding phase I trial [33]. In this context, the study design of dose escalation trials usually includes patients who are allocated in different cohorts and have received different dose levels in order to identify the maximum tolerate dose (MTD). It is a common practice to expand the number of patients recruited in the MTD cohort to explore activity and extend the evaluation of safety results. The inclusion of a NI hypothesis in the superiority-based analysis of this extended MTD cohort will help to rank the activity data with the other endpoints. In accordance, we planned a single-stage, single-arm A'hern design [23]. We accepted the same constraints which were reported in the previous examples, except for type II error (20%):

(p0, p1, α, 1 ‒ β, NIM) = (0.20, 0.45, 0.1, 0.2, 1.2) (21)

Among 19 patients recruited, we will achieve a positive finding in the NI and superiority analyses with 6 (31.5%) and 7 responders (36.8%), respectively [23]. In addition to evaluate the clinical pharmacokinetic profile of the drug, a phase I study offers the opportunity to test the prespecified pharmacodynamic and biomarker hypotheses that were obtained in previous pre-clinical studies [34,35]. The aim of these phase I studies is to enrich the data obtained in early setting, that in turn will allow the study design improvement of further randomized trials [[35], [36], [37]]. In accordance with our example, if data suggest both a good tolerability with a positive result in the NI analysis, a well-characterized pharmacokinetic profile, and a novel target in molecular pathway, the randomized trial could be designed combining experimental therapy with previous standard of care. Alternatively, the experimental therapy will be evaluated as monotherapy in further randomized clinical trials if an equivalent toxic profile compared with previous example is obtained, with a positive result in superiority analysis, and a similar molecular target respect to standard of care. Finally, a vey toxic treatment with a higher efficacy than standard of care, or a non-toxic treatment without a clinically meaningfully activity will not be tested in a new randomized clinical trials if it had a negative risk-benefit ratio [38,39]. So, the isolated assessment of activity, safety, pharmacokinetics, or pharmacodynamic data would have no sense. In order to combine these data in a single evaluation, it is important to grade qualitatively the intensity of clinical activity. Thus, analyzing both NI and superiority objectives is more informative than only consider one of them separately [40,41].

## 4 Discussion

Single-arm phase II trials are considered the first efficacy screenings of new investigational agents in humans. Additionally, these trials are important milestones towards testing safety and adapting the biomarker strategies validated in preclinical stages [34]. Although the NI question can be relevant in the phase II setting [5], it is not usually considered on designing single-arm clinical trials during early clinical development [42,43]. Previous forays into precision medicine have shown that identifying correctly both the target population and potential predictive biomarkers may be more critical for treatment success than simple demonstration of superior efficacy against an alternative [36,37,44]. The use of biomarker information in clinical trials has great potential for efficiently identifying patients most helped by specific treatments [45,46]. These biomarkers must have proven their clinical validity in prospective, randomized trials with a superiority design in the enriched population [47]. Consequently, designing a proof-of-concept phase II study that permits NI and superiority analyses will allow for more informed decisions that rank the magnitude of clinical activity and other parameters, such as safety, pharmacokinetic, and pharmacodynamic data [7]. As NI analysis is allowed, it could be assumed that this design facilitates that ineffective therapies were evaluated in a randomized study. However, this only could be true if we evaluated the treatment exclusively based on the NI result, forgetting the other objectives. This makes no sense, because any new drug development plan performs a risk-benefit assessment weighing all the endpoints [38]. In accordance, a new therapy non-inferior to the standard of care with the same safety profile, and biological target will not be much promising. On the contrary, a new therapy with a good safety profile, that demonstrates to be non-inferior to the standard of care would be a promising companion if it targets a different biological pathway compared with the standard therapy [48].

Previous studies have proposed different strategies to evaluate multiple endpoints (efficacy and toxicity) in phase I and II single arm trials. EFFTox, Gumbel model, or continual reassessment method adaptations for Bayesian perspective, or single-stage binomial trials and Simon two-stage adaptations for frequentist paradigm. However, these designs have been planned to evaluate the superiority of the new drug activity among a theorical or historical reference. They not considered to compare non-inferiority and superiority hypothesis to grade the magnitude of clinical activity in the early clinical stages [[49], [50], [51], [52]].

A prior systematic review, which aimed to evaluate the characteristics of phase II trials that best predict for phase III outcome, selected 270 single-arm phase II studies between 1981 and 2012. All these selected studies led to a phase III clinical trial. The meta-analysis showed that 168 single-arm trials were not positive, while 61 (36.3%) achieved a positive phase III results despite not having obtained a positive result in the proof-of-concept study [36]. Additionally, Jardin DL and colleagues reported that 10% of FDA anti-cancer drugs approvals between January 2009 and June 2014 obtained a negative result in prior phase II clinical trials [53]. In a number of these studies, despite the low response rates, clinical activity was considered not worse than conventional treatments and allowing to continue with Phase III trials according to other parameters, such as the prolonged duration of clinical benefit or the positive safety profile [25,[29], [30], [31]]. Overall, these data demonstrate that combining the superiority and NI analyses is useful to rank the intensity of clinical activity with other relevant parameters. This strategy of analysis of phase II trials has enriched the design of successful phase III leading to the marketing authorization of the product from regulatory authorities [53]. This suggests that the NI question is relevant in the phase II setting and it is not so rare. However, the NI hypothesis was not planned, and it was only considered to deal with negative findings in proof-of-concept trials. This strategy leads to an increase in the probability of type I error and the number of false positives [6]. In accordance, both systematic reviews reported and high rate of negative phase III trials after a negative phase II superiority trial (between 64% and 85%) [36,53]. However, if the decision to include a NI analysis had been preplanned and included in the statistical design, investigators would have additional information about magnitude of response (non-inferior or superior) or other parameters without a type I error inflation. In addition, the NI comparison with an historical control has probed its validity identifying subgroups of patients in adjuvant setting who can avoid the toxic effects of chemotherapy [26].

The most popular design for phase II cancer clinical trials is a single-arm, two-stage Simon's design. Numerous extensions have been proposed for the Simon's design, including randomized multi-arm trials designed to select the winner among the proposed therapeutics (Pick-the-winner study design) [54]. However, the inference procedures used in two-stage designs are often not corrected to account for the adaptive nature of these designs. A maximum likelihood estimator of the RR, the number of positive responses/total number of patients, is biased. CI and p-value should not be computed as if the data were obtained in a single stage due to the possibility of early termination [10]. Different methods have been proposed to obtain a proper inference from the Simon's two-stage design [10]. The use of the UMVUE to estimate RR is recommended as it addresses situations when the actual number of patients recruited is equal to or different from preplanned values [27]. The calculation of RR, p-values and CIs has been incorporated in open-access statistical libraries (packages “clinfun” and “OneArmPhaseTwoStudy”, R statistical software) based on previously published methods [11,28].

We observed some differences between calculated (p-value) and simulated values (type I error). However, they were not relevant to the most common design constraints used in phase II single-arm trials (0.01–0.1 type I errors and 0.1 to 0.2 type II errors). Using the UMVUE-based calculation method, we proved that the same level of agreement between calculated and simulated values (type I and II errors) results from both two-stage Simon's superiority designs and designs in which NI analysis was allowed. Our findings suggest that the proposed method for analyzing NI in superiority-based designs did not introduce a bias under the most usual constrains for alpha and beta errors. So, our results recommend that single-arm study designs can be planned to attain the usual levels of statistical error (alpha and beta errors equal or lower than 0.2). In study designs with error rates which are higher than usual levels, it would be necessary to simulate alpha and beta errors to confirm that statistical errors are attained at desired level.

The major limitations of this method are based on bringing together the inherent complexities of a study with historical controls and a NI analysis. However, these limitations are quite common in comparative study designs of NI, because NIM must be established based on historical controls evidence [6]. Additionally, some bias (e.g. as selection of inappropriate patients, poor compliance and insufficient follow-up, that can lead to erroneously conclude that a treatment is not inferior to placebo in a comparative study) go against a positive achievement when the comparison is done among a theoretical rate of efficacy deduced from historical controls. As comparative designs of NI, to declare a therapy as non-inferior in a single-arm trial, we need to demonstrate assay sensitivity based on an adequate trial design and conduct [12]. In accordance, lower sample sizes in phase II single-arm trials are not more challenging for NI analyses respect to superiority ones if trial is properly preplanned and conducted. Our results suggest that we can conserve the same levels of alpha and beta errors after including the NI analysis without increase sample size.

Furthermore, the NI analysis with the UMVUE-based calculation method may be extended to two-stage designs with both futility and superiority boundaries (as outlined in the Supplementary Methods) [55]. We limited our results to two-stage designs that are most popularly for phase II cancer clinical trials, but the methods discussed in this article could be extended to phase II trials with any number of stages [9]. Likewise, we can design a single-arm time-to-event study with a NI hypothesis based on the exponential maximum likelihood estimator, one-sample log-rank tests or other approximations to the Kaplan-Meyer estimations [[55], [56], [57]]. If we assume the same number of patients as in superiority analysis, we would formulate the NI rejection hazard rate (λ_0ni_) from the hazard rate assumed under H_0_ in the superiority analysis (λ_0_) and the NIM estimated from historical studies [6] (λ_0NI_ = λ_0_/NIM).

Including a NI analysis in superiority-based clinical trials, may be useful for weighing additional factors such as clinical benefit duration, safety, cost, or biomarker strategy while assessing activity and early efficacy rate. The results of previous single-arm designs leading to a successful phase III trials or identifying subgroups of patients who can avoid the toxic effects of chemotherapy suggest that NI question is relevant in non-comparative studies. Implementation of this strategy can be achieved through simple adaptations to the Simon's two-stage design and other existing designs for one-arm phase II clinical trials.

## Declaration of competing interest

We wish to draw the attention of the Editor to the following facts which may be considered as potential conflicts of interest and to significant financial contributions to this work:

Miguel Sampayo-Cordero reports personal fees from Hospital Vall d’Hebron, Roche, Nestle Health Science, Laboratorios Leti, Medica Scientia Innovation Research (MedSIR), Syntax for Science, Ability Pharma and Scienco Klinico, outside the submitted work.

Bernat Miguel-Huguet declares no conflict of interest.

José Pérez-García has received consulting and advisor fees from: Roche and Eli Lilly.

David Páez declares a scientific advisory role for Amgen, Sanofi, Merck Serono, F. Hoffmann-La Roche Ltd, Lilly and Servier.

Ángel L. Guerrero-Zotano declares no conflict of interest.

Javier Garde Noguera declares no conflict of interest.

Elena Aguirre has received consulting and advisor fees from Pfizer and honorarias from: Roche, Novartis, Celgene, Eisai and Pfizer.

Esther Holgado declares no conflict of interest.

Elena López-Miranda declares no conflict of interest.

Xin Huang is a statistician at Pfizer Oncology.

Antonio Llombart-Cussac has received consulting and advisor fees from Roche, GlaxoSmithKline, Novartis, Celgene, Eisai, and AstraZeneca and has stock options, patents and intellectual property from MedSIR.

Andrea Malfettone declares no conflict of interest.

Javier Cortés has received consulting and advisor fees from: Roche, Celgene, Cellestia, AstraZeneca, Biothera Pharmaceutical, Merus, Seattle Genetics, Daiichi Sankyo and Erytech. In addition, Javier Cortés has received honorarias from: Roche, Novartis, Celgene, Eisai, Pfizer and Samsung. Add more, Javier Cortés has received research funding fees to the institution from Roche. Finally, Javier Cortés has stock options, patents and intellectual property from MedSIR.
