# Supplementary material for: Inclusion of non-inferiority analysis in superiority-based clinical trials with single-arm, two-stage Simon's design
Source: Contemp Clin Trials Commun. 2020 Nov 28;20:100678. doi: 10.1016/j.conctc.2020.100678 (PMC7733004; doi:10.1016/j.conctc.2020.100678)
Supplement: Multimedia component 1 [file mmc1.docx]

**SUPPLEMENTARY METHODS**

**Inclusion of non-inferiority analysis in superiority-based clinical trials with single-arm, two-stage Simon’s design**

Miguel Sampayo-Cordero, Bernat Miguel-Huguet, José Pérez-García, David Páez, Angel L. Guerrero-Zotano, Javier Garde Noguera, Elena Aguirre, Esther Holgado, Elena López-Miranda, Xin Huang, Antonio Llombart-Cussac, Andrea Malfettone, Javier Cortés

**List of abbreviations:** CI: confidence interval; NI: non-inferiority; NIM: non-inferiority margin; RR: response rate; UMVUE: Uniformly Minimum Variance Unbiased Estimator.

1. **One-stage design with binary outcomes**

The hypotheses of a superiority analysis about the true response rate (RR) (p) for a single arm design are:

H_0_: p ≤ p_0_, (eq. 1)

H_1_: p ≥ p_1_, (eq. 2)

where “p_0_” represents the pre-specified fixed null response probability, which can be given by the historical RR of an active control treatment; and “p_1_” is the minimum desired response probability required to progress the treatment to a subsequent trial phase.[1]

For a specified type I error rate α, the rejection value α is defined as the smallest number satisfying the following:

B(a|n, p_0_) ≥ 1- α (eq. 3)

And the power should be calculated by:

1 - β = 1 - B(a|n, p_1_) (eq. 4)

where “a” is the expected number of responding patients, “n” is the expected number of patients included in the study and “s” represents the observed number of responding patients. The cumulative distribution function and the probability mass function (PMF) are defined as follows:

B(s|n, p) = $\sum_{i=0}^{s} b(i|n,p)$; (eq. 5)

and

b(s|n,p) = $\left( \begin{matrix} n \\ s \end{matrix} \right)$p^s^ (1 - p)^n-s^ (eq. 6)

respectively.[2]

Accordingly, the study will achieve a positive finding when “p” will be higher than “p_0_” and when significance level evaluated by binomial test will be ≤ α. Although “p” may not be higher than p_1_.

1. **Non-inferiority (NI) analysis**

The goal of NI analysis is to show that the effect of the test compound (p) is not inferior to the effect of the active control (p_0_) by a specified amount, also known as the NI margin (NIM). The null and alternative hypotheses should be defined as follows [3]:

H_0_: p_0_ – p ≥ NIM (p is inferior to the control (p_0_) by NIM or more); (eq. 7)

H_a_: p_0_ – p < NIM (p is inferior to the control (p_0_) by less than NIM). (eq. 8)

Although the NI margin used in a trial can be no higher than the entire assumed effect of the active control against placebo (M1), it is generally desirable to choose a lower margin (M2) that reflects the largest loss of effect clinically acceptable [3]. Showing NI to M1 provides assurance that the test drug had an effect greater than zero, but in many cases that is sufficient to conclude that the test drug had a clinically acceptable effect [3]. In a fixed margin approach the NIM could be estimated as risk ratio or risk difference, through the average effect of the active control over placebo in historical studies [(p _control_ / p _placebo_) > 1 or (p _control_ - p _placebo_) > 0], for example:

Relative risk = 2.64, 95% confidence interval (CI): (1.72 to 3.56). (eq. 9)

Risk difference = 0.15, 95% CI: (0.07 to 0.22). (eq. 10)

We selected the 95% CI lower bound (1.72 or 0.07) and adjusted to retain at least 50% of the historical effect of active control versus placebo arms ([1.72^(1-0.5) = 1.31] or [0.07*(1-0.5) = 0.035]) [3]. Accordingly, the NIM calculated describes a ratio or a difference reflecting the largest loss of effect in control group RR (p_0_) considered clinically acceptable. Therefore, the null and alternative hypothesis of NI analysis can be defined as follows and depending on p_0_/NIM:

H_0_: p ≤ (p_0_ / NIM_as ratio_) or H_0_: p ≤ (p_0_ - NIM_as difference_); (eq. 11)

H_1_: p > (p_0_ / NIM_as ratio_) or H_1_: p > (p_0_ - NIM_as difference_); (eq. 12)

Risk ratio is preferred because it is less affected than risk differences by variability in the event rates of the placebo group.[3]

1. **Include a NI analyses in a superiority based single-arm design**

In a superiority analysis design with tumor response as the primary endpoint, analyze firstly a NI hypothesis does not inflate the type I error rate when NI analysis and NIM are properly pre-specified [4]. Additionally, the final number of responders needed to achieve the NI objective will always equal or lower than the prespecified superiority efficacy boundary (a). We assumed the same number of patients as superiority analysis (n); and “a_ni_” (number of responding patients in NI analysis) is chosen as the highest integer satisfying the type I error rate in NI analysis (α_ni_) ≤ α.

B(a_ni_|n, p_0_ / NIM) ≥ 1- α (eq. 13)

The power should be calculated as:

1 - β_ni_ = B(a_ni_ -1|n, p_0_/NIM) (eq. 14)

where

a_ni_ ≤ a; 1 - β ≤ 1 - β_ni_. (eq. 15)

Accordingly, the study achieves a positive finding when “p” is equal to or higher than “p_0_ / NIM” and significance levels evaluated by binomial test in NI analysis are ≤ α. As the NI analysis has the same expected accrual and lower or equal number of responders needed to declare significance than superiority analysis (a_ni_ ≤ a), power always will be equal or greater in NI than superiority criteria. Thus, this design can assess superiority and NI criteria with the same sample size, type I and type II error rates used in the superiority strategy. Thus, this design evaluates superiority and NI criteria with the same sample size and type I and type II error rates used in the superiority strategy.

1. **Two-stage design**

A two-stage design is defined by the number of patients to be treated during stages one and two (M = 1 and M = 2), n_1_ and n_2_; the expected number of patients to accept (≤a_1_) or reject (≥b_1_) null hypothesis at stage one and the expected number of patients to declare superiority at final analysis (>a), where (a_1_ <b_1_ < a). So, we specify them by:

[(a_1_,b_1_) / n_1_, a / n], (eq. 16)

where n= n_1_ + n_2_ is the maximal sample size. The values of [(a_1_,b_1_) / n_1_, a / n] were determined based on pre-specified design parameters (p_0_, p_1_, α_1_, 1-β) as in single-stage design cases [2]. Final analysis depends on the cumulative number of responding patients observed (s) by the stopping stage (i.e. s = s_1_ if M = 1 and s = s_1_+ s_2_ if M = 2) and on the number of patients accrued during the study stages 1 (n_1_) and 2 (n_2_).

Considering that for K = 1, 2, X_k_ were independent B(n_k_,p) random variables, the probability of rejecting the treatment (or equivalent failing to reject H_0_: p ≤ p_0_) for two-stage design is expressed as:

R(p) = B(a_1_|n_1_,p) + $\sum_{x=a1+1}^{J} b\left( s | n1,p \right)B(a-s|n2,p)$ (eq. 17)

where J = min(n_1_,a) in a two-stage design with only an stage one futility boundary (Simon’s design); and J = (b_1_-1) for a two-stage design with both futility and efficacy boundaries.

When the true response is p, the constraints on type I error probability and power are expressed as:

R(p_0_) ≥ 1-α and R(p_1_) ≤ β. (eq. 18)

Given (p_0_, p_1_, α_1_, 1-β), there are many two-stage designs [(a_1_/b_1_)/n_1_, a/n] satisfying the constraints with an upper limit for n, usually between 0.85 and 1.5 times the sample size for a single stage design. The probability of concluding the study early, PET(p), is the first term in Eq.(1). B(a_1_|n_1_,p), and the expected sample size for this design, E(N|p), is n_1_PET(p) + n (1 − PET(p)). In a previous work, Simon proposed two criteria for selecting a good two-stage design among these designs [5]. The minimax design minimizes the maximum sample size “n” satisfying (α, 1 - β)-constraint. On the other hand, the so-called optimal design minimizes the expected sample size EN under the null hypothesis.

The most popular estimator of RR p for (M, S) = (m, s) is the sample proportion. For example:

s/n_1_ if m=1

$\bar{p}$= (eq. 19)

s/(n_1_+n_2_) if m=2

The so-called maximum likelihood estimator (MLE) is always negatively biased for standard two-stage trials with futility stopping only.[6] For (M, S) = (m, s), the Uniformly Minimum Variance Unbiased Estimator (UMVUE) of p for two-stage phase II trials is given by:

s/n_1_ if m=1

$\bar{p}$= (Eq. 20)

$\frac{\sum_{x1=(a1+1) \vee(s-n2)}^{s \wedge(b1-1)} \left( \begin{matrix} n1-1 \\ x1-1 \end{matrix} \right)\left( \begin{matrix} n2 \\ s-x1 \end{matrix} \right)}{\sum_{x1=(a1+1) \vee(s-n2)}^{s \wedge(b1-1} \left( \begin{matrix} n1 \\ x1 \end{matrix} \right)\left( \begin{matrix} n2 \\ s-x1 \end{matrix} \right)}$ if m=2

where

“a ∧ b = min(a, b)”;

“a ∨ b = max(a, b)”;

“x! = x× (x ‒ 1) × … × 2 × 1 ([7])”;

“b_1_ = n_1_+1” for Simon’s two-stage designs and “b_1_” represents the expected number of patients for rejecting “H_0_” at stage one if a two-stage design with both futility and efficacy boundaries.

It is interesting to remark that the UMVUE and the MLE are identical if the trial stops after stage one, (i.e. m = 1). For a true RR of p, the probability mass function (PMF) of (M, S), f(m, s|p) = Pr(M = m, S=s), is given as [7]:

p^s^(1-p)^n1-s^ $\left( \begin{matrix} n1 \\ s \end{matrix} \right)$ if m=1, 0 ≤ s ≤ a_1_ or b_1_ or b=1,_1_

f(m,s|p) = (Eq. 21)

p^s^(1-p)^n1+n2-s^ $\sum_{x1=(a1+1)}^{s \wedge(b1-1} \left( \begin{matrix} n1 \\ x1 \end{matrix} \right)\left( \begin{matrix} n2 \\ s-x1 \end{matrix} \right)$ if m=2, a_1_+1 s ≤ b_1_ – 1+ n_2_

Since the UMVUE of p is a function of (M, S), its PMF is derived from f(m, s|p).

Through a phase II trial, we intend to conduct a statistical test to reject or accept its therapeutic potential. If we reject or fail to reject the null hypothesis, we should be able to provide a p-value as a measure of how much evidence the decision is based on against the null hypothesis.

By using the stochastic ordering of the UMVUE, Jung *et al.* proposed a p-value method for two-stage phase II clinical trials . A p-value is defined as the probability of observing an extreme test statistic value toward the direction of H1 when H0 is true, so they propose calculating the probability of observing a UMVUE value larger than that obtained from the study under H0. Let p˜ denote the UMVUE for the RR observed in a two-stage phase II trial specified by (a_1_, b_1_, a, n_1_, n_2_). Given (M, S) = (m, s), p-value = Pr{p˜ (M, S) ≥ p˜ (m, s)|p_0_} based on UMVUE can be calculated as follows:

$\sum_{j=s}^{n1} f\left( 1,j|p0 \right)$ if m = 1, s_1_

p-value= 1- $\sum_{j=0}^{s-1} f\left( 1,j|p0 \right)$ if m = 1, s_1_ (eq. 22)

$\sum_{j=b1}^{n1} f\left( 1,j|p0 \right)+\sum_{j=s}^{b1-1+n2} f\left( 2,j|p0 \right)$ if m = 2

UMVUE-based calculation of p-value does not require specification of the critical values at the terminal stage. It can be used to test H_0_: p = 0 against H_1_: p > p_0_ based on the pre-specified type I error rate α when the realized sample size is different from that specified in the design at the stopping stage (M1 or M2). Additionally, power may be calculated with UMVUE-based calculation for p_1_.

In accordance with previous explanations in single-stage studies, we can include a NI analysis in superiority based clinical trial provided that NI analysis and NIM must be pre-specified properly) ([4]). We should assume the same (a_1_, b_1_, n_1_ and n) as superiority analysis and substitute p_0_ by p_0_ / NIM in equations 20, 21, and 22. The s_ni_ is chosen as the lowest integer satisfying α_ni_ ≤ α. Where s_ni_ ≤ a and 1 - β_ni_ ≤ 1 - β.

1. **Implementation**

A user-defined function has been written in R software [8] (additional files) to calculate point estimator, p-value, and power in two-stage designs with a binary outcome, according to the UMVUE method [7]. R “Clinfun” library (function “ph2simon”)[9] was used to find the optimal and minimax two-stage Simon’s designs (a_1_, n_1_/a, n) under specific constraints (p_0_, p_1_, α and 1 ‒ β). We calculated all possible p_0_ ranging between 0.05 to 0.95 in 0.05 increments. We assumed in all designs a 0.15 percentage difference with p_1_. Six scenarios have been assumed in accordance with type I and II error constraints. The type I error values were 0.1, 0.05, and 0.01 and the type II error values were 0.2 and 0.1. For each design, we calculated the lower number of responding patients at final analysis (a_ni_), required to declare the NI hypothesis to be statistically significant for type I and II errors assumed in superiority design. The NIMs selected to formulate the rejection proportion (p0_ni_ = p_0_ / NIM) ranged between 1.15 to 1.45 in 0.05 increments.[10] Moreover, we consider the NIM with value of 1 to include the designs where superiority analysis was not include the NI analysis. A total of 12,768 two-stage Simon’s optimal and minimax designs were computed.

Simulation was used to calculate probability of type I (alpha) and II (beta) errors in every design under the number of events selected at final analysis (a_ni_). We generated binomial random samples (function “Rbinom”) based on (a_1_,n_1_/a,n) (additional files). Additionally, we calculated the number of random samples generated (NSim) needed to attain a 95% confidence (z_(0,025)_) that simulated values of alpha and beta errors (E) are within 0.5% of true values [11]. Therefore, we accepted maximum differences between calculated and simulated values (alpha and beta errors), which ranged from 0.095 to 0.0105. The random seed was computed using R function “sample.int(.Machine$integer.max, 1)” and was the integer 1440679596.

According to the [11]:

NSim = (Z_α/2_* 100* sd / (E * y))^2 (eq. 23)

where “sd” and “y” represent standard deviation and sample average of type I and II errors with 30 simulations, respectively.

Eighty thousand (80,000 ≈ 74,861) and eigthy-five thousand (85,000 ≈ 83,015) random samples were run for type I and II errors, respectively.

Agreement between calculated and simulated values was analyzed with Bland-Altman plots. We plotted the differences between calculated and simulated scores (calculated-simulated) against the average of calculated and simulated scores (calculated + simulated) / 2. The 95% limits of agreement were calculated with traditional methods or the V-shaped procedure if proportional bias was detected between the two measures. Finally, we presented minimum and maximum differences observed between values, because the latter was easier to interpret in terms of clinically acceptable limits [12,13].

**REFERENCES**

[1] R.P. A’Hern, Sample size tables for exact single-stage phase II designs, Stat Med. 20 (2001) 859–866. https://doi.org/10.1002/sim.721.

[2] Sin-Ho Jung, Randomized Phase II Cancer Clinical Trials., in: Chapman and Hall/CRC, 2013.

[3] U.S. Department of Health and Human Services Food and Drug Administration, Center for Drug Evaluation and Research (CDER), Center for Biologics Evaluation and Research (CBER), Non-Inferiority Clinical Trials to Establish Effectiveness. Guidance for Industry., Https://Www.Fda.Gov/Downloads/Drugs/Guidances/UCM202140.Pdf. (2016).

[4] Committee For Proprietary medicinal products (CPMP), The European Agency for the Evaluation of Medicinal Products, Points to consider on switching between superiority and non-inferiority., Http://Www.Ema.Europa.Eu/Docs/En_GB/Document_library/Scientific_guideline/2009/09/WC500003658.Pdf. (2000).

[5] R. Simon, Optimal two-stage designs for phase II clinical trials, Control Clin Trials. 10 (1989) 1–10.

[6] S.-H. Jung, K.M. Kim, On the estimation of the binomial probability in multistage clinical trials, Stat Med. 23 (2004) 881–896. https://doi.org/10.1002/sim.1653.

[7] S.-H. Jung, Statistical issues for design and analysis of single-arm multi-stage phase II cancer clinical trials, Contemp Clin Trials. 42 (2015) 9–17. https://doi.org/10.1016/j.cct.2015.02.007.

[8] R.C. Team, R: A language and environment for statistical computing. Vienna, Austria: R Foundation for Statistical Computing., Https://Www.R-Project.Org/. (2016).

[9] Venkatraman E. Seshan, Package “clinfun” - Clinical Trial Design and Data Analysis Functions. Version 1.0.14, Https://Cran.r-Project.Org/Web/Packages/Clinfun/Clinfun.Pdf. (2017).

[10] S. Tanaka, Y. Kinjo, Y. Kataoka, K. Yoshimura, S. Teramukai, Statistical issues and recommendations for noninferiority trials in oncology: a systematic review, Clin. Cancer Res. 18 (2012) 1837–1847. https://doi.org/10.1158/1078-0432.CCR-11-1653.

[11] William Oberle, Weapons and Materials Research Directorate, ARL, Monte Carlo Simulations: Number of Iterations and Accuracy, Www.Dtic.Mil/Get-Tr-Doc/Pdf?AD=ADA621501. (2015).

[12] H. Pottel, Critical review of method comparison studies for the evaluation of estimating glomerular filtration rate equations, Int J Nephrol Kidney Failure. Volume 1 (2015). http://dx.doi.org/10.16966/ ijnkf.102.

[13] J. Ludbrook, Confidence in Altman-Bland plots: a critical review of the method of differences, Clin. Exp. Pharmacol. Physiol. 37 (2010) 143–149. https://doi.org/10.1111/j.1440-1681.2009.05288.x.
