# Supplementary material for: Inclusion of non-inferiority analysis in superiority-based clinical trials with single-arm, two-stage Simon's design
Source: Contemp Clin Trials Commun. 2020 Nov 28;20:100678. doi: 10.1016/j.conctc.2020.100678 (PMC7733004; doi:10.1016/j.conctc.2020.100678)
Supplement: Multimedia component 2 [file mmc2.zip › 3_Sampayo-Cordero M, et al_CCT_SupplementaryDataSummary.docx]

**Article Title**

Dataset in support of the article “Inclusion of non-inferiority analysis in superiority-based clinical trials with single-arm, two-stage Simon’s design”.

**Authors**

Miguel Sampayo-Cordero, ^1^ Bernat Miguel-Huguet, ^2^ José Pérez-García, ^1,3^ David Páez, ^1,4^ Ángel L. Guerrero-Zotano, ^5^ Javier Garde Noguera, ^6^ Elena Aguirre, ^7^ Esther Holgado, ^1,8^ Elena López-Miranda, ^1,8^ Xin Huang, ^9^ Antonio Llombart-Cussac, ^1,10^ Andrea Malfettone, ^1^ Javier Cortés, ^1,3,12^

^1^ Medica Scientia Innovation Research (MedSIR), Barcelona, Spain

^2^ Hospital Universitari de Bellvitge, L'Hospitalet, Barcelona, Spain

^3^ IOB, Institute of Oncology, QuironSalud Group, Barcelona and Madrid, Spain

^4^ Hospital de la Santa Creu i Sant Pau, Universitat Autònoma de Barcelona, Barcelona, Spain

^5^ Vanderbilt-Ingram Cancer Center; Vanderbilt University Medical Center, Nashville, Tennessee, USA

^6^ Hospital Arnau de Vilanova, Valencia, Spain

^7^ Hospital QuironSalud Group, Zaragoza, Spain

^8^ Ramón y Cajal University Hospital, Madrid, Spain

^9^ Pfizer Global Research and Development, La Jolla, USA

^10^ FISABIO - Hospital Arnau de Vilanova, Valencia, Spain

^12^ Vall d´Hebron Institute of Oncology (VHIO), Barcelona, Spain

**Corresponding author:** Miguel Sampayo-Cordero, Medica Scientia Innovation Research (MedSIR), Rambla de Catalunya 2, 2D – 08007 Barcelona, Spain. E-mail: [sampayo.mc@gmail.com](mailto:sampayo.mc@gmail.com). Telephone: +34 932 214 135

**ABSTRACT**

The non-inferiority (NI) hypothesis is not usually considered in the early phases of clinical development. A proof-of-concept phase II study that allows for the analysis of NI, if superiority criteria cannot be met, may balance between multiple endpoints and additional parameters, which will result in more informed decisions in regard to the therapeutics’ potential value.

A total of 12,768 two-stage Simon’s design trials were constructed based on different assumptions of rejection response probability, minimum desired response probability, type I and II errors, and NI margins. P-value and type II error were calculated with stochastic ordering using Uniformly Minimum Variance Unbiased Estimator. Type I and II errors were simulated using the Monte Carlo method. Agreement between calculated and simulated values was analyzed with Bland-Altman plots.

For further interpretation of the data presented in this article, please see the research article “Inclusion of non-inferiority analysis in superiority-based clinical trials with single-arm, two-stage Simon’s design” (Sampayo-Cordero M et al., 2020) [1].

**Key Words:** Clinical trial; Non-inferiority; Switching to non-inferiority; Two-stage; Single-arm; Phase II; Early stopping; Group sequential designs; Adaptive designs; Non-comparative.

**Specifications Table**

| **Subject** | Single-arm two-stage Phase II cancer trial designs with tumor response as the primary endpoint. |
| --- | --- |
| **Specific subject area** | Switching between superiority to non-inferiority in a single-arm design. |
| **Type of data** | Three tables and two figures |
| **How data were acquired** | Computed based on different design assumptions about the effect of a hypothetical test drug (p1), an active control (p1), alpha error (α), power (1 – β) and non-inferiority margin. The package “Clinfun” (function “ph2simon”) from R software was used for computing these designs.^2, 3^ |
| **Data format** | -Three tables in “csv” format: Each row in the tables, except the top row, describes a Simon two-stage design. Columns describe characteristics of these designs.  - Two R code files: We attach the R code used to simulate “csv” tables and plot figures 1 and 2: |
| **Parameters for data collection** | A user-defined function has been written in R software to calculate point estimator, p-value, and power in two-stage designs with a binary outcome, according stochastic ordering using uniformly minimum variance unbiased estimator method.^2^  Simulation was used to calculate probability of type I (alpha) and II (beta) errors in every design under the number of events selected at final analysis (ani). We generated binomial random samples (R function “Rbinom”). ^2^  The random seed was computed using R function “sample.int(.Machine$integer.max, 1)” and was the integer 1440679596. ^2^ |
| **Description of data collection** | Agreement between calculated and simulated values was analyzed with Bland-Altman plots. We plotted the differences between calculated and simulated scores (calculated-simulated) against the average of calculated and simulated scores (calculated + simulated) / 2. The 95% limits of agreement were calculated with traditional methods or the V-shaped procedure if proportional bias was detected between the two measures. Finally, we presented minimum and maximum differences observed between values, because the latter was easier to interpret in terms of clinically acceptable limits.^3, 4^ |
| **Data source location** | Institution: Medica Scentia Innovation Research (MedSIR).  City/Town/Region: Barcelona.  Country: Spain |
| **Data accessibility** | File attached with the article  • 3_Sampayo-CorderoM_SupplementalDataset_0.csv  • 3_Sampayo-CorderoM_SupplementalDataset_1.csv  • 3_Sampayo-CorderoM_SupplementalDataset_2.csv  • 3_Sampayo-Cordero M, et al_Supplemental File1.R  • 3_Sampayo-Cordero M, et al_Supplemental File2.R |
| **Related research article** | Author’s name: Miguel Sampayo-Cordero  Title: Inclusion of non-inferiority analysis in superiority-based clinical trials with single-arm, two-stage Simon’s design ^1^.  Journal: Contemporary Clinical Trials  DOI: … |

**Value of the Data**

- The data was used to evaluate the validity of a non-inferiority analysis in superiority-based clinical trials with single-Arm, two-stage simon’s design.
- The analysis proposed was based on stochastic ordering using uniformly minimum variance unbiased estimator.
- We analyze the agreement between calculated p-values and power and simulated type I and II errors under different design constraints and non-inferiority margin assumptions.
- In accordance, we evaluated if we could conserve the same design constraints of alpha and beta errors after switching to non-inferiority analysis without increase the sample size.
- Switching to NI analysis when the superiority criteria are not reached may balance between multiple efficacy endpoints and additional parameters (safety, biomarker strategy…). The development of this strategy can be achieved by straightforward adaptations to the existing statistical designs of one-arm phase II trials.

**Data**

In addition to the main manuscript, we attached five supplementary documents as follows:

- 3_Sampayo-CorderoM_SupplementalDataset_0.csv
- 3_Sampayo-CorderoM_SupplementalDataset_1.csv
- 3_Sampayo-CorderoM_SupplementalDataset_2.csv
- 3_Sampayo-Cordero M, et al_Supplemental File1.R
- 3_Sampayo-Cordero M, et al_Supplemental File2.R

Comma separated values (CSV):

The three tables of commas separated values (CSV) describe characteristics of the different Simon two-stage designs (“3_Sampayo-CorderoM_SupplementalDataset_0”, “3_Sampayo-CorderoM_SupplementalDataset_1” and “3_Sampayo-CorderoM_SupplementalDataset_2”). Each row in the tables, except the top row, describes a Simon two-stage design. There are 12.768 two-stage designs in each table. The columns describe:

1. The effect of an active control drug measured as percentage of responders (p0),
2. The desired effect of a test group measured as percentage of responders (p1),
3. Planned superiority design (a1/n1, a/n) described as number of responders at first stage to move second stage (a1), first stage sample size (n1), number of final analysis responders needed to achieve a positive result in superiority analysis (a) and final analysis sample size (n). [1]
4. The number of responders at final analysis needed to achieve a positive result in non-inferiority analysis (H0_reject.NI). The p-value of the non-inferiority analysis when the number of responders to obtain a positive finding are achieved (ani). The p-value is calculated with stochastic ordering using uniformly minimum variance unbiased estimator (alphaEq). [1]
5. The expected power of the non-inferiority design assuming a fixed number of responders to obtain a positive finding (a_ni_). The power is calculated with stochastic ordering using uniformly minimum variance unbiased estimator (powEq).
6. The alpha error of the non-inferiority design simulated with Monte Carlo method. The binomial random samples were generated with R function “Rbinom” (alphaSim).[2]
7. The statistical power of the non-inferiority design simulated with Monte Carlo method. The binomial random samples were generated with R function “Rbinom” (powSim). [2]
8. The non-inferiority margin prespecified in the design (NIM).[3,4]
9. The type of Simon two-stage design simulated: optimal and minimax (Optimal). [5]

In “3_Sampayo-CorderoM_SupplementalDataset_0” the number of random samples generated in each design to calculate simulated values were 30. In “3_Sampayo-CorderoM_SupplementalDataset_1” the number of random samples generated in each design to calculate simulated values were 80,000. In “3_Sampayo-CorderoM_SupplementalDataset_2” the number of random samples generated in each design to calculate simulated values were 85,000.

We use information of “3_Sampayo-CorderoM_SupplementalDataset_0” to calculate the mean and the standard deviation of the samples of type I and II errors. We introduce this values in equation 1 (see next section) to estimate the number of random samples needed to attain a 95% confidence that simulated values of alpha and beta errors (ɛ) are within 0.5% of true values.

We plot the information of “3_Sampayo-CorderoM_SupplementalDataset_1” table on figure 1 and information of “3_Sampayo-CorderoM_SupplementalDataset_2” on figure 2. Figure 1 calculate the agreement between calculated p-value and simulated alpha error. Figure 2 calculated the agreement between calculated power and simulated (1-beta error). The agreement was calculated in accordance with Bland-Altman methods. We plotted the differences between calculated and simulated scores (calculated-simulated) against the average of calculated and simulated scores (calculated + simulated) / 2. The 95% limits of agreement were calculated with traditional methods or the V-shaped procedure if proportional bias was detected between the two measures. Finally, we presented minimum and maximum differences observed between values, because the latter was easier to interpret in terms of clinically acceptable limits*.*[6,7]

R code files:

We attach the R code used to generate supplemental tables and figures 1 and 2:

- 3_Sampayo-Cordero M, et al_Supplemental File1: This file develops two user defined functions: “UMVUEpvalueSIMON” and “simulate”. The first is used to calculate p-value and power based on stochastic ordering using uniformly minimum variance unbiased estimator method. The second is used to simulate alpha and (1-beta) errors based on Monte Carlo method.

- 3_Sampayo-Cordero M, et al_Supplemental File2: The first section of this file (“Loop to develop all data tables”) develops the loop used to generate the two supplemental tables based on the different constraint of Simon’s two-stage designs (maximum sample size, p0, p1, alfa and beta errors and optimal or minimax design), Monte Carlo method (random seed and number of random samples) and non-inferiority analysis (non-inferiority margin).

The second section of this file (“Number of random samples needed”) calculates the number of random samples needed to attain a 95% confidence that simulated values of alpha and beta errors (ɛ) are within 0.5% of true values.

The third section of this file (“Plotting results”) develops the coded needed to plot the results of supplemental tables 1 and 2 in figures 1 and 2.

# Figure 1. Agreement between calculated p-value and simulated alpha errors in Simon’s two-stage clinical designs.

# Absolute differences have been plotted against average of calculated and simulated scores. The type I errors values considered were 0.1, 0.05 and 0.01, and type II errors values were 0.2 and 0.1. The NIMs selected to formulate the rejection proportion (p_0ni_ = p_0_ / NIM) were (1, 1.15, 1.2, 1.25, 1.30, 1.35, 1.4, and 1.45). A maximum of 2.5% deviation defined the 95% limits of agreement.


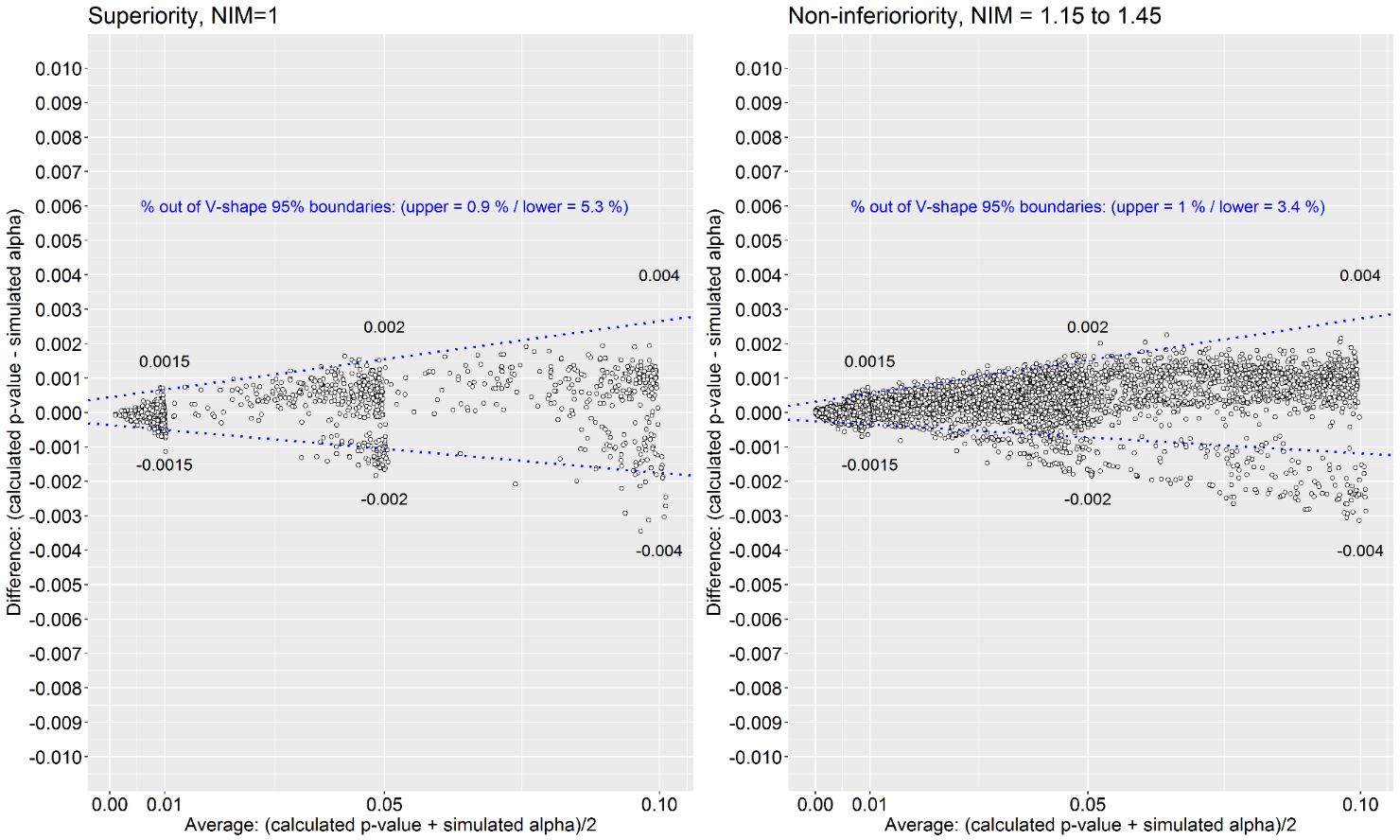


# Figure 2. Agreement between calculated and simulated type II error in Simon’s two-stage clinical designs.

# Absolute differences have been plotted against average of calculated and simulated scores. The type I values considered were 0.1, 0.05 and 0.01, and type II errors were 0.2 and 0.1. The NIMs selected to formulate the rejection proportion (p0ni = p0/NIM) were (1, 1.15, 1.2, 1.25, 1.30, 1.35, 1.4 and 1.45). A maximum of 2.5% deviation defined the 95% limits of agreement.


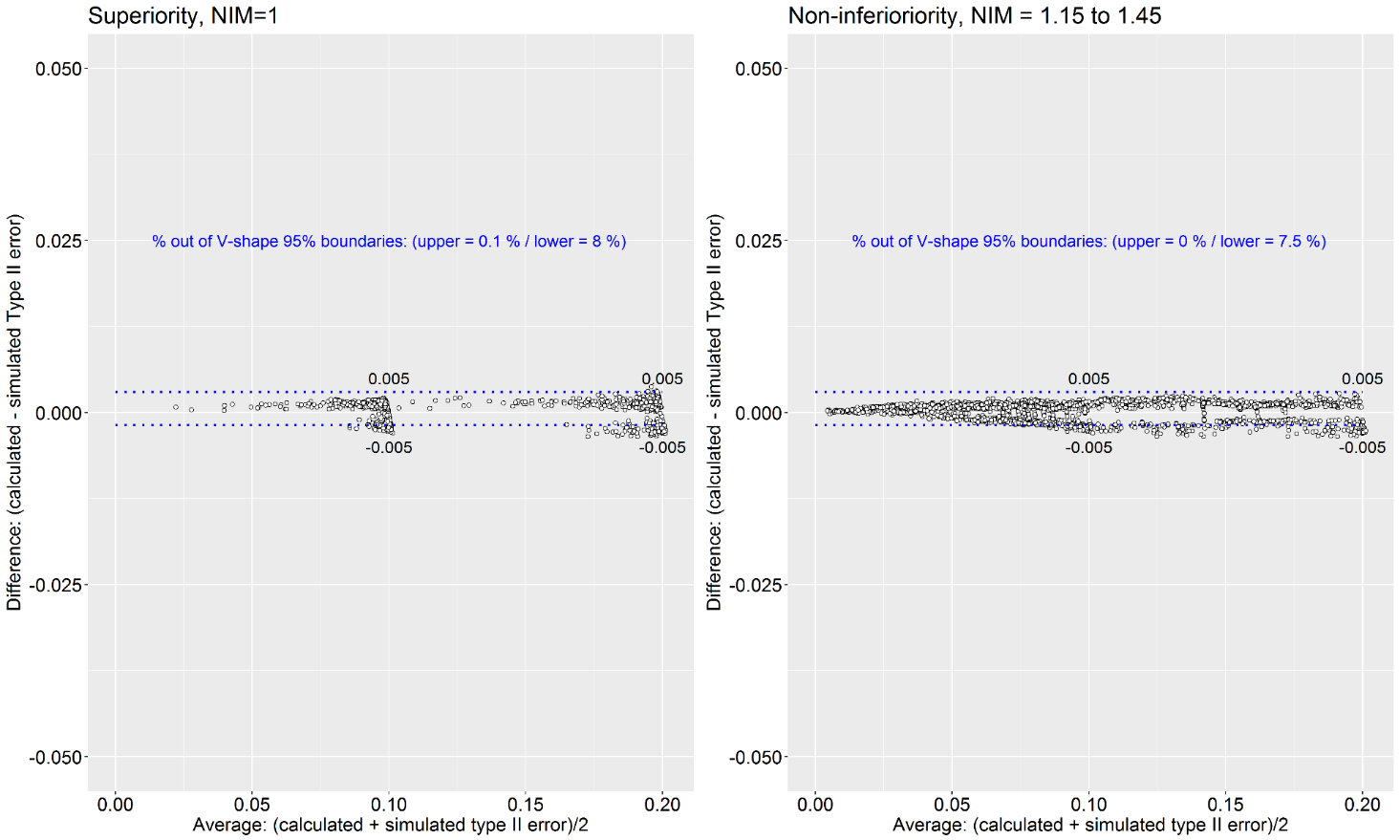


**REFERENCES**

1. Sampayo-Cordero M, Miguel-Huguet B, 2 Pérez-García J, Páez D, Guerrero-Zotano A, Garde-Noguera J, Aguirre E, Holgado E, López-Miranda E, Huang X, Llombart-Cussac A, Malfettone A, Cortés J, Inclusion of non-inferiority analysis in superiority-based clinical trials with single-arm, two-stage Simon’s design, Contemp. Clin. Trials (pending acceptance).

[1] Sin-Ho Jung: Randomized Phase II Cancer Clinical Trials., in Chapman and Hall/CRC. 2013, (n.d.).

[2] Team RC., R: A language and environment for statistical computing., R Foundation for Statistical Computing, Vienna, Austria, 2016. https://www.R-project.org/. 2016.

[3] U.S. Department of Health and Human Services Food and Drug Administration, Center for Drug Evaluation and Research (CDER), Center for Biologics Evaluation and Research (CBER): Non-Inferiority Clinical Trials to Establish Effectiveness. Guidance for Industry. https://www.fda.gov/downloads/Drugs/Guidances/UCM202140.pdf , 2016, (n.d.).

[4] Committee For Proprietary medicinal products (CPMP), The European Agency for the Evaluation of Medicinal Products: Points to consider on switching between superiority and non-inferiority. http://www.ema.europa.eu/docs/en_GB/document_library/Scientific_guideline/2009/09/WC500003658.pdf , 2000, (n.d.).

[5] Venkatraman E. Seshan, .Package “clinfun” - Clinical Trial Design and Data Analysis Functions., 2017. https://cran.r-project.org/web/packages/clinfun/clinfun.pdf.

[6] G. Omuse, D. Maina, J. Mwangi, C. Wambua, A. Kanyua, E. Kagotho, A. Amayo, P. Ojwang, R. Erasmus, Comparison of equations for estimating glomerular filtration rate in screening for chronic kidney disease in asymptomatic black Africans: a cross sectional study, BMC Nephrol. 18 (2017). https://doi.org/10.1186/s12882-017-0788-y.

[7] J. Ludbrook, Confidence in Altman-Bland plots: a critical review of the method of differences, Clin. Exp. Pharmacol. Physiol. 37 (2010) 143–149. https://doi.org/10.1111/j.1440-1681.2009.05288.x.
